# Buckling of Microtubules on a 2D Elastic Medium

**DOI:** 10.1038/srep17222

**Published:** 2015-11-24

**Authors:** Arif Md. Rashedul Kabir, Daisuke Inoue, Tanjina Afrin, Hiroyuki Mayama, Kazuki Sada, Akira Kakugo

**Affiliations:** 1 Faculty of Science, Hokkaido University, Sapporo 060-0810, Japan; 2 Graduate School of Chemical Sciences and Engineering, Hokkaido University, Sapporo 060-0810, Japan; 3 Department of Chemistry, Asahikawa Medical University, Asahikawa 078-8510, Japan

## Abstract

We have demonstrated compression stress induced mechanical deformation of microtubules (MTs) on a two-dimensional elastic medium and investigated the role of compression strain, strain rate, and a MT-associated protein in the deformation of MTs. We show that MTs, supported on a two-dimensional substrate by a MT-associated protein kinesin, undergo buckling when they are subjected to compression stress. Compression strain strongly affects the extent of buckling, although compression rate has no substantial effect on the buckling of MTs. Most importantly, the density of kinesin is found to play the key role in determining the buckling mode of MTs. We have made a comparison between our experimental results and the ‘elastic foundation model’ that theoretically predicts the buckling behavior of MTs and its connection to MT-associated proteins. Taking into consideration the role of kinesin in altering the mechanical property of MTs, we are able to explain the buckling behavior of MTs by the elastic foundation model. This work will help understand the buckling mechanism of MTs and its connection to MT-associated proteins or surrounding medium, and consequently will aid in obtaining a meticulous scenario of the compression stress induced deformation of MTs in cells.

Cells depend on the cytoskeleton for successful completion of its various spatial and mechanical functions such as growth, development, migration, etc. which are crucial to survival of living organisms^1^,^2^,^3^. Participation of cytoskeletal components in mechanical stimulation of cells is significantly important with respect to the mechano-responsiveness of cells that triggers a variety of physiological functions of the cells^4^. Microtubules, the most rigid filamentous component of the cytoskeleton^1^,^5^ with a large slenderness ratio^6^ ( > 100), play pivotal roles in the mechano-responsiveness of cells. MTs actively participate in cell contractility^4^, development and maintenance of cell polarity^7^, and also in a number of cellular activities such as intracellular transport, regulation of cell morphology and cell mechanics^1^,^5^,^8^,^9^. While MTs are engaged in such activities in cell, they are often found curved or buckled^10^,^11^,^12^,^13^,^14^,^15^,^16^ in response to internally generated force, e.g. force due to constrained MT polymerization^17^,^18^,^19^, interaction with motor proteins^20^, actomyosin contractility^16^,^17^,^21^, or external mechanical impact^22^,^23^,^24^. Generally a slender rod, when subjected to axial compression, undergoes classical Euler buckling, i.e. buckling with long wavelength, which is accounted for by the ‘elastic column model’^25^. However, unlike the classical Euler buckling, compression stress induced deformation of slender MT filaments in living cells has been manifested by short wavelength multiwave buckling mode^10^,^12^,^16^,^26^,^27^. Based on theoretical and computational studies, reinforcement of the MTs by surrounding elastic medium or MT-associated proteins around the MTs in cells was suspected to play the key role in making the difference in buckling behavior between MTs and an isolated slender rod^10^,^28^,^29^,^30^. The ‘elastic foundation model’, which is based on the buckling of MTs on a two-dimensional (2D) elastic medium, has been a simplified but useful approach for predicting the buckling behavior of MTs and accounting the role of MT-associated proteins in the buckling of MTs^28^,^29^,^30^,^31^,^32^. However, no experimental evidence has been available so far that may help verify the theoretical predictions on buckling of MTs and role of MT-associated proteins in this regard. In this work, by using a newly developed experimental setup, we have demonstrated the first ever buckling of MTs on a 2D elastic medium and experimentally investigated the role of compression strain, strain rate and a MT-associated protein ‘kinesin’ in the buckling of MTs. By comparing with our experimental results, we then verified the theoretical predictions of the elastic foundation model on MT buckling. Here, first we investigated the response of MTs, laterally supported on an elastic substrate through interaction with the MT-associated protein kinesin, to axially applied compression stress. MTs were found to undergo mechanical deformation, i.e. buckling when they were subjected to the compression stress. Extent of buckling of MTs was dependent on the applied compression strain, although no considerable effect of strain rate on MT buckling was noticed. Next, we investigated the role of kinesin in the buckling of MTs; the density of the kinesin or in other words, the spacing between kinesins worked as a key parameter in determining the buckling mode of MTs. MTs showed long wavelength Eulerian buckling at low kinesin density (at long kinesin spacing); whereas short wavelength multiwave uniform buckling of MTs, reminiscent to the *in vivo* buckling mode of MTs, was observed at high kinesin density (at short kinesin spacing). Consequent comparison of our experimental results with the predictions of the elastic foundation model suggested that modulation of mechanical property of MTs by any MT-associated protein is required to take into account while considering the role of surrounding medium or MT-associated proteins in buckling of MTs. This work is expected to offer a new insight in the understanding of compression stress induced buckling of MTs, and would help confirm the role of MT-associated proteins or surrounding medium in determining the buckling behavior of MTs, which consequently will aid in obtaining a comprehensive scenario of the compression stress induced deformation of MTs in cells.

## Methods

### Preparation of tubulin and kinesin

Tubulin was purified from porcine brain using a high-concentration PIPES buffer (1 M PIPES, 20 mM EGTA, 10 mM MgCl_2_; pH adjusted to 6.8 using KOH). High-molarity PIPES buffer (HMPB) and BRB80 buffer were prepared using PIPES from Sigma, and the pH was adjusted using KOH^33^. Green fluorescent protein(GFP)-fused kinesin-1 consisting of the first 560 amino acid residue of human kinesin-1 (K560-GFP) was prepared by partially modifying previously reported expression and purification methods^34^.

### Preparation of labeled tubulin and MTs

Rhodamine-labelled tubulin was prepared using 5/6-carboxy-tetramethyl-rhodamine succinimidyl ester (TAMRA-SE; Invitrogen) according to the standard technique^35^ and the labeling ratio was 1.0 as determined by measuring the absorbance of the protein at 280 nm and that of tetramethyl-rhodamine at 555 nm. Rhodamine-labelled MTs were obtained by polymerizing a mixture of rhodamine tubulin (RT) and non-labelled tubulin (WT) at 37 °C (RT: WT = 4:1; final tubulin concentration = 55.6 μM). The solution containing the MTs was then diluted with motility buffer (80 mM PIPES, 1 mM EGTA, 2 mM MgCl_2_, 0.5 mg mL^−1^ casein, 1 mM DTT, 10 μM paclitaxel; pH 6.8).

### Compression tests of MTs

Compression tests of MTs were demonstrated using a newly developed mechanical chamber. The mechanical chamber consists of two main parts: cover plate and base plate where the baseplate contains a computer controlled stretcher/compressor. First, a small piece of PDMS with dimension 4.0 × 5.0 × 0.05 mm^3^ (L × W × T) (elastic modulus: ~1.86 MPa; Fuso Rubber Industry Co. Ltd.) was fixed horizontally at the stretcher of the mechanical chamber, and elongated 100% through tension by using the computer controlled stretcher. At this stage, the length of the elongated PDMS substrate was 8.0 mm. A narrow region on the top surface of the elongated PDMS was then plasma treated for 3 min (10 Pa, 8 mA) by a plasma etcher (SEDE-GE; Meiwafosis Co. Ltd.) to increase its hydrophilicity in order to use it as a flowcell. Next anti-GFP antibody (Invitrogen) of a concentration of 0.1 mg mL^−1^ (5 μL) was applied to the plasma treated PDMS surface (flowcell). 10 μL of K560-GFP solution of prescribed concentrations, e.g. 10, 30, 50, 100 and 200 nM (~80 mM PIPES, ~40 mM NaCl, 1 mM EGTA, 1 mM MgCl_2_, 1 mM DTT, 10 μM paclitaxel; pH 6.8) was introduced in respective case and incubated for 3 min to bind the kinesins to the antibody. The flowcell was washed with 10 μL of motility buffer (~80 mM PIPES, 1 mM EGTA, 1 mM MgCl_2_, 0.5 mg mL^−1^ casein, 1 mM DTT, 10 μM paclitaxel; pH 6.8). Next, 10 μL of 100 nM MT solution was introduced and incubated for 3 min, followed by washing with 20 μL of motility buffer. In this work, MTs were deposited on the PDMS substrate via interaction with the kinesins in the absence of any nucleotide. MTs were labeled with a fluorescent dye in order to monitor the response of MTs to the applied compression stress under a fluorescence microscope. During flowcell preparation, shear flow was used by applying MT solution from one end of the narrow flowcell to align MTs parallel to the stretch axis, so that compression stress could be applied along the longitudinal axis of MT filaments. After preparation of flow cell on PDMS, the mechanical chamber was closed and humid nitrogen gas was kept passing through the chamber to remove oxygen from the chamber. After passing the nitrogen gas for one hour, the chamber was mounted to the stage of a fluorescence microscope. Finally the tensile stress at the elongated PDMS was released through compression that consequently produced compression stress at the MTs attached to the PDMS substrate. All the aforementioned experiments were performed at room temperature.

### Determination of kinesin density on PDMS substrate

Kinesin solutions of a wide range of concentrations were applied to the gold-coated crystals of a quartz crystal microbalance (QCM), prior to which surface of QCM sensor was coated with anti-GFP antibody in each case. Using ‘Sauerbrey equation’ density of kinesin deposited on the gold coated QCM crystal was determined for each of the kinesin concentrations. Next, after applying anti-GFP antibody, kinesin solutions of the same concentrations were applied to the surface of a gold coated cover glass and fluorescence intensity of kinesins were collected for every kinesin concentration. A standard curve was prepared from which we obtained a linear correlation factor between fluorescence intensity and kinesin density as: (kinesin density)/(fluorescence intensity) = 27.4 molecule·μm^−2^·au^−1^ (au stands for arbitrary unit of fluorescence intensity). Using the standard curve (the linear correlation factor) and fluorescence intensity, kinesin density on PDMS substrate of the compression tests were determined for different kinesin concentrations.

### Microscopic image capture and image analyses

Samples were illuminated with a 100 W mercury lamp and visualized by using an epi-fluorescence microscope (Eclipse Ti; Nikon) using an oil-coupled Plan Apo 60 × 1.40 objective (Nikon). Filter blocks with UV-cut specification (TRITC: EX540/25, DM565, BA606/55; GFP-HQ: EX455-485, DM495, BA500-545; Nikon) were used in the optical path of the microscope that allowed the visualization of samples but eliminated the UV part of radiation and minimized the harmful effect of UV radiation on samples. Images were captured using a cooled CMOS camera (Neo sCMOS; Andor) connected to a PC. To capture a field of view for more than several minutes, ND filters (ND4, 25% transmittance) were inserted into the illuminating light path of the fluorescence microscope to avoid photobleaching.

### Measurement of applied strain, buckling wavelength, amplitude and radius of curvature

In this work the term ‘strain’ is specified with respect to the compression strain applied to the PDMS substrate. Here, for measurement of strain, length of the elongated PDMS (8.0 mm) was considered as the initial length and all subsequently applied strains were quantified with respect to this length of the PDMS (8.0 mm). Images of MTs captured by fluorescence microscope were analyzed using the image analysis software ‘Image J’. The length of MTs considered for investigating their buckling behavior ranged from ~20 μm to ~50 μm. Only the MTs aligned parallel to the compression axis were considered for analyses. For quantifying the deformation of MTs, buckling wavelength and amplitude were manually measured from the fluorescence microscopy images of deformed MTs, using the image analysis software. Brightness and contrast of MTs were also adjusted using the image analysis software. The radius of curvature of a deformed MT was calculated using the following equation (1) and equation (2):


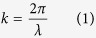



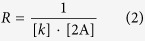


Here, *k* is wave number, *λ* is buckling wavelength, *R* is radius of curvature and A is buckling amplitude.

### Selection of buckling wavelength for verification of elastic foundation model

In order to compare the experimental results with the predictions by the elastic foundation model, we selected the wavelength of buckled MTs by considering the dependence of buckling wavelength on applied strain. As mentioned in the ‘results and discussion’ section, buckling wavelength of MTs was found strongly dependent on the applied strain. When the applied strain was increased gradually from a low value, buckling wavelength decreased, which is much prominent at relatively low kinesin concentrations (e.g., 10 nM). Initially, at relatively low strain region buckling wavelength of MTs sharply decreased on increasing the strain, and this continued till ~15% strain, which we term as ‘transitional strain’; after which the change of wavelength on further increasing the strain was much smaller compared to that observed at the low strain region. A difference in steepness in the change in wavelength is noticeable below and above this transitional strain as shown in Supplementary Information (Fig. S1). We roughly considered the wavelength corresponding to this transitional strain (i.e., wavelength corresponding to the intersection of the two straight lines shown in Fig. S1) and plotted this wavelength against different kinesin spacing obtained for different kinesin concentrations. Alternatively we also used the critical buckling wavelength i.e., wavelength at the critical strain, for verification of the elastic foundation model.

## Results and Discussion

**Figure 1:**
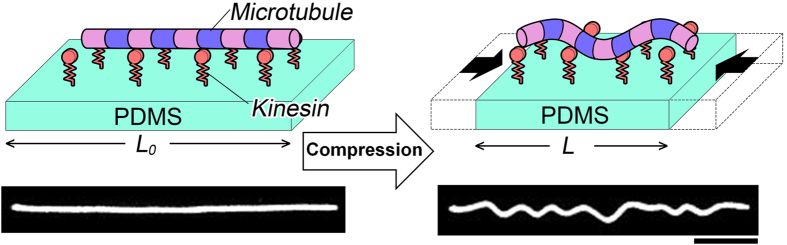
Experimental design used to demonstrate the buckling of MTs on a 2D elastic medium. Schematic illustrations and representative fluorescence microscopy images show how the compression stress applied at the PDMS substrate caused buckling of the MT attached to the substrate through interaction with kinesin. The fluorescence microscopy images were captured for the kinesin concentration of 50 nM and the image of buckled MT was captured at 25.0% strain. Compression stress was applied at a rate of 0.42% s^−1^. *L*_*0*_ and *L* represent the length of PDMS substrate before and after compression respectively. For simplicity, the anti-GFP antibody is not shown in the schematic illustrations. Scale bar: 10 μm.

**Figure 2:**
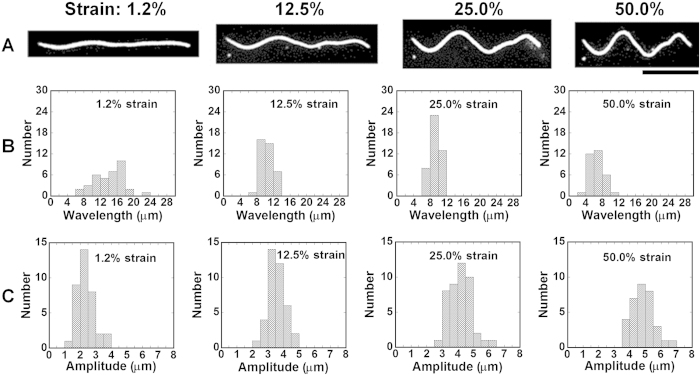
Effect of compression strain on the buckling behavior of MT. (**A**) Fluorescence microscopy images show how buckling of a MT develops with the increase in compression strain. The images were captured for the kinesin concentration of 50 nM and the compression rate was 0.42% s^−1^. The histograms clearly show the change in the buckling wavelength (**B**), and buckling amplitude (**C**) on increasing the compression strain. Scale bar: 10 μm.

To demonstrate the compression stress induced deformation of MTs on a 2D elastic medium and investigate the role of the MT-associated protein kinesin in the deformation of MTs, we have developed an experimental setup named ‘mechanical chamber system’ which is shown in Supplementary Information (Fig. S2). Using the mechanical chamber, a soft elastomeric substrate, e.g. polydimethylsiloxane (PDMS) was subjected to compression, which consequently developed compression stress at the MTs deposited on the PDMS substrate surface and resulted in their mechanical deformation i.e., buckling, as shown schematically in the Fig. 1. In this work the kinesins adsorbed to the PDMS substrate worked as linear springs, and were aligned on the same plane and fixed perpendicular to the MTs that were deposited at the PDMS surface through interaction with the kinesins. Kinesins were distributed along the MTs with one end permanently fixed to the MTs and the other end to the PDMS substrate via interaction with anti GFP-antibody. Compression of the PDMS substrate then developed compression stress at the MTs deposited to the substrate, which resulted in mechanical deformation of the MTs manifested by buckling, as shown by the fluorescence microscopy images in Fig. 1. From the fluorescence microscopy image of buckled MTs, the buckling of MTs under the present experimental condition appears reminiscent to the uniform multiwave mode, rather than the localized buckling mode^32^. Therefore, this experimental system turns out to realize the practical demonstration of laterally constrained MTs with ‘both ends clamped’ condition^32^. At the onset of MT buckling at relatively low level of compression strain, buckling crests or change in curvature along the MT filaments was found to develop. The compression strain at which the first buckling crest or curvature change was observed is termed hereafter as ‘critical strain’. To characterize the compression stress induced buckling of MTs, we have monitored the wavelength and amplitude of buckled MTs. As shown in the Fig. 2A, a MT filament produced the first buckling crest when the applied strain reached the critical strain; ~1.2% strain for the presented case of arbitrarily fixed kinesin concentration (50 nM). Although the first buckling crest appeared at a certain strain i.e., at the critical strain, all subsequently applied strains were found to bring changes in buckling wavelength and amplitude of the deformed MTs. On increasing the strain beyond the critical strain the buckling wavelength was found to decrease, whereas the buckling amplitude increased (Fig. 2A). These observations suggest that buckling of MTs under compression stress may not be a discrete phenomenon but a continuous process, once the critical strain is reached. Distribution of the buckling wavelength and amplitude of deformed MTs at different strain values are shown in Fig. 2B,C respectively (number of MT considered for analysis, n = 30). The histograms clearly reveal that on increasing the strain, distribution of buckling wavelength gradually became narrower and shifted towards lower values but the distribution of buckling amplitude followed an opposite trend.

**Figure 3:**
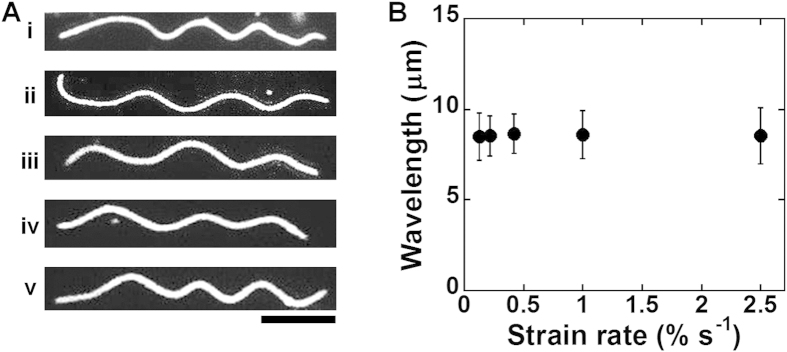
Role of compression rate in the buckling of MTs. (**A**) Fluorescence microscopy images show the effect of strain rate on the compression stress induced buckling of MTs. Compression tests of MTs were performed at the strain rate of: i) 0.13, ii) 0.22, iii) 0.42, iv) 1.0 and v) 2.5% s^−1^. In these experiments the kinesin concentration was fixed at 50 nM and the fluorescence microscopy images were captured after applying 25.0% strain. (**B**) Strain rate has no considerable effect on the buckling of MTs. Error bar: standard deviation; scale bar: 10 μm.

Next, we investigated the effect of strain rate on the compression stress induced buckling of MTs, since the strain rate is one of the considerable variables in mechanical deformation of materials^36^. Keeping the kinesin concentration same to that used in the already discussed experiment above, i.e. 50 nM the compression tests of MTs were conducted at different strain rates. Here by using the computer controlled compressor of the mechanical chamber we varied the strain rate over one order of magnitude, such as from 0.13% s^−1^ to 2.5% s^−1^, which is the maximum range of strain rate that could be applied by using the mechanical chamber. Changes in buckling wavelength of deformed MTs to changes in the strain rate were evaluated. We found no considerable effect of strain rate on the buckling of MTs under the present experimental conditions, which is shown in Fig. 3, although during deformation biopolymers are susceptible to respond to any change in strain rate due to their viscoelastic property. Similar insensitivity to the strain rate was previously reported also in case of tensile stress induced deformation of MTs and composite materials^36^,^37^,^38^,^39^.

Now, in order to investigate the role of the MT-associated protein kinesin in the buckling of MTs, which at the same time will allow verification of the elastic foundation model, we have demonstrated buckling of MTs on the 2D elastic medium by varying the concentration of kinesin over a wide range i.e., from 10 to 200 nM. Increase in the concentration of kinesin was found to increase the fluorescence intensity on the PDMS substrate surface, which is shown in Supplementary Information (Fig. S3). Increase in fluorescence intensity confirms that density of kinesin on the PDMS substrate was increased when kinesins of higher concentrations were applied to the PDMS surface. By using quartz crystal microbalance (QCM) we have estimated the kinesin density^37^ and the spacing between kinesins on PDMS substrate for different concentrations of applied kinesin. For the 10, 30, 50, 100, and 200 nM kinesin concentration, the kinesin density on the PDMS substrate was found to be approximately 112, 381, 749, 1800, and 2982 kinesins/μm^2^, which in turn resulted in a kinesin spacing of 95, 51, 36, 23, and 18 nm respectively. The inter-kinesin distance (kinesin spacing) estimated by QCM could be supported by the atomic force microscopy image of kinesins on the PDMS substrate which is available in Supplementary Information (Fig. S4). Here, it is to note that, we further increased the kinesin concentration to 300 nM which resulted in a density of ~4125 kinesins/μm^2^. This density of kinesin is close to the saturation density of kinesins reported in literature^40^, although this saturation density also depends on the substrate^41^. We performed compression test of MTs at this kinesin concentration and as a result we found the buckling crests of the deformed MTs were very small and closely spaced as shown in the Supplementary Information (Fig. S5). Sometimes the buckling crests were difficult to trace which rendered them unsuitable for any quantitative characterization. Therefore, results of compression tests of MTs obtained using the 300 nM kinesin could not be considered further.

**Figure 4:**
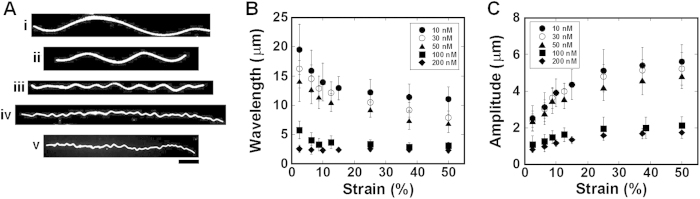
Buckling of MTs at different kinesin concentrations. (**A**) Representative fluorescence microscopy images show the effect of kinesin concentration on the buckling of MTs. Images were captured at 12.5% strain and the kinesin concentrations were: i) 10, ii) 30, iii) 50, iv) 100 and v) 200 nM that resulted in kinesin spacing of 95, 51, 36, 23, and 18 nm respectively. Compression stress was applied at a rate of 0.42% s^−1^. To clearly show the buckling wavelength and amplitude the brightness and contrast of images of buckled MTs were adjusted, particularly for the cases of 100 and 200 nM kinesin concentrations where the buckling wavelength and amplitude were very small and the buckling crests were closely spaced. (**B**) Change of buckling wavelength and (**C**) buckling amplitude with the change of applied compression strain at different kinesin concentrations. Error bar: standard deviation; scale bar: 5 μm.

Effect of strain on the buckling of MTs (buckling wavelength and amplitude) is shown in Fig. 4, where fixing the strain rate at 0.42% s^−1^ we also varied the concentration of kinesin. At the lowest kinesin concentration i.e., at 10 nM, the first buckling crest was noticed at ~0.6% strain and at this kinesin concentration no additional buckling crest along the MT filaments was developed even at higher strains. At this lowest kinesin concentration and at the critical strain, the average buckling wavelength and amplitude were 21.14 ± 5.6 μm and 2.16 ± 0.89 μm respectively. On increasing the kinesin concentration e.g., at the highest concentration of 200 nM, the critical strain increased to ~2.0%. At this kinesin concentration, additional buckling crests (in addition to that noticed at the critical strain) were found to develop occasionally on increasing the strain beyond the critical strain. At the critical strain, average buckling wavelength and amplitude were 3.11 ± 1.21 μm and 0.72 ± 0.16 μm respectively when the kinesin concentration was the highest (200 nM). Therefore, concentration of kinesin was found to strongly affect the buckling extent of MTs which is evident from the above differences in critical strain, critical buckling wavelength and amplitude of deformed MTs at different kinesin concentrations. As shown in the Fig. 4, with the increase in kinesin concentration wavelength and amplitude of buckled MTs decreased which is valid for any of the applied strains. On the other hand, for a fixed kinesin concentration, with the increase in applied strain the buckling wavelength was found to decrease and buckling amplitude increased substantially when the kinesin concentration was relatively low (e.g., 10 nM). However, for a relatively higher kinesin concentration (e.g., 200 nM), changes in wavelength and amplitude with the change of strain were not that significant compared to that observed for low kinesin concentrations. Here, an important manifestation of the effect of kinesin concentration (kinesin density or spacing) on MT buckling is the transition of buckling mode of MTs. When the kinesin concentration was low (e.g., 10 nM), MTs showed long wavelength Eulerian buckling (Fig. 4A (i)), similar to that reported for most of the cases of slender rods^25^ and *in vitro* buckling of isolated MTs^6^,^19^,^20^,^42^,^43^,^44^,^45^. On the other hand, when the kinesin concentration was gradually increased (e.g., 200 nM), the buckling mode of MTs was changed from long wavelength Eulerian to uniform short wavelength multiwave buckling mode (Fig. 4A (v)), which is reminiscent to the *in vivo* buckling mode of MTs^10^,^12^,^16^,^26^,^27^. Thus, kinesin’s density or spacing dependent transition of MT’s buckling mode observed in our experiments, which could never be observed in the previous *in vitro* studies on MT buckling, suggests that MT-associated proteins or surrounding medium might play a key role in determining the buckling mode of MTs.

We also monitored the radius of curvature of buckled MTs at different kinesin concentrations, result of which is shown in Supplementary Information (Fig. S6). Similar to the buckling wavelength, and amplitude, the radius of curvature of buckled MTs was substantially dependent on the kinesin concentration. The radius of curvature of MTs was relatively larger at low kinesin concentrations which decreased with the increase in strain. For example, for the 10 nM kinesin concentration, the radius of curvature of MTs was 4.18 ± 1.69 μm at 2.5% strain, which decreased to 0.54 ± 0.25 μm at 50% strain. In contrast, at 200 nM kinesin concentration, the radii of curvature were 0.23 ± 0.10 μm and 0.09 ± 0.03 μm at 2.5% and 50% strain respectively. Waterman-Storer et al. reported buckling induced breakage of MTs in cell which occurred when the radius of curvature of buckled MTs was 0.6 ± 0.15 μm^17^. In our work, we achieved such low radius of curvature at relatively higher kinesin concentrations (e.g., 100 or 200 nM) and sometimes we also noticed breakage of buckled MTs at those kinesin concentrations, as shown in the Supplementary Information (Fig. S7), although it was difficult to confirm particularly at which radius of curvature the buckled MTs underwent fracturing. In contrary to the cases of high kinesin concentrations, no such breakage of MTs was observed for the kinesin concentrations lower than 100 nM within the range of strain studied, though the radii of curvature became small at relatively higher strains. To confirm further, we exposed such buckled MTs to adenosine triphosphate (ATP). As the result, which is shown in the Supplementary Information (Fig. S7), we found the buckled MTs started gliding on the kinesin carpet, adopted a straight morphology soon and their buckling was disappeared over time. Here, using the radius of curvature (*R*) of deformed MTs, the bending energy *E*_bend_ stored in the MTs^46^ at different experimental conditions can be calculated by using equation (3)-


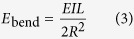


Here, *E* is the Young’s modulus, and *I* is the second moment of inertia of MT and *L* is the contour length of MT along a single buckling crest. For the lowest kinesin concentration (10 nM), the bending energy stored in the MTs were (0.47 pN)*L* and (27.99 pN)*L* at 2.5% and 50% strain respectively. For 200 nM kinesin, the bending energy were (23.97 pN)*L* and (156.87 pN)*L* at 2.5% and 50% strain respectively. We measured the average contour length of MTs along a single buckling crest (*L*) which was ~27.5 ± 5.2 μm and 2.8 ± 0.9 μm for 10 nM and 200 nM kinesin respectively. Using the measured *L* we calculated the bending energy (*E*_bend_) stored in the deformed MTs at different strains for two kinesin concentrations (10 and 200 nM), which is shown in Supplementary Information (Fig. S8). Here, we considered *I* = 32.82 × 10^−32^ m^4^; and *E* = 49.75 and 7.15 MPa for 10 and 200 nM kinesin respectively^37^. From the Fig. S8, it appears that under compression MTs behave more elastically at low kinesin concentration (10 nM) than at high kinesin concentration (200 nM); modulation of MT’s mechanical property by kinesin might also be involved here. Moreover, observed fracturing of buckled MTs at high kinesin concentration (Fig. S7) might be a reason why the bending energy stored in buckled MTs at 200 nM kinesin did not increase continuously for all the strain values as observed for the case of 10 nM kinesin.

Now, for the buckling of MTs supported by a continuum elastic foundation the buckling wavelength, 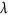 and critical buckling force, 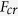 could be expressed by equation (4) and equation (5) respectively^30^,^32^.


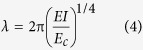


and,


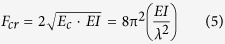


Here, *E* is the Young’s modulus and *EI* is the bending rigidity of MT and *E*_*c*_ is the elastic-foundation modulus. The elastic foundation-modulus, *E*_*c*_ is correlated to the spring constant, *k* and kinesin spacing, *L*_*d*_ as shown by equation (6)^32^-


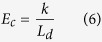


Then, from equation (4) and equation (6) we get equation (7) and equation (8) as follows-


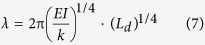


and,


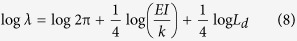


**Figure 5:**
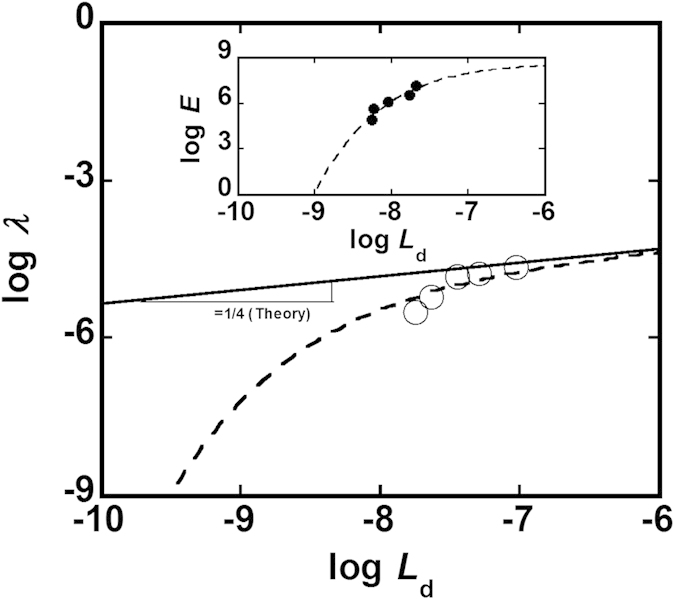
Comparison between theory and experimental results on buckling of MTs and its dependence on kinesin spacing. The theory (elastic foundation model) is verified using the experimental results on the buckling of MTs obtained in this work. Inset shows the curve fitting of the dependence of MT’s Young’s modulus (*E*) on kinesin spacing (*L*_d_). The solid line in the main panel (slope = 1/4) is drawn to show the relationship between kinesin spacing and buckling wavelength as theoretically predicted by the elastic foundation model. The dashed line is the fitting curve according to the modified elastic foundation model based on the present work that takes into account the change in Young’s modulus of MT by kinesins.

Therefore, plot of 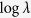 against 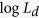 using the data obtained from our experiments allows the verification of the elastic foundation model. To verify the elastic foundation model, we have selected the buckling wavelength at critical strain and also by considering the effect of strain on MT buckling for different kinesin concentrations (see ‘Methods’ and Fig. S1 for detail). As shown in the Supplementary Information (Fig. S9), at the long kinesin spacing region the slope of the straight line drawn following our experimental data points was in an excellent agreement with that predicted theoretically by the elastic foundation model. On the other hand, a considerable deviation of the experimental results from the theoretical prediction was noticed at relatively short kinesin spacing region. This result suggest that, at least another factor might be involved in this regard, which was not taken into account by the elastic foundation model so far and therefore it failed to satisfactorily correspond to the experimental results at all the kinesin spacing studied in this work. To investigate further, here we particularly focus on the interaction between the MT-associated protein kinesin and the MTs as it has been suspected on several occasions that MT-associated proteins can alter the mechanical property of MTs^47^,^48^,^49^,^50^. Recently evolving evidences have just started to confirm the role of MT-associated proteins in modulating the mechanical property of MTs^51^. In our recent work, we experimentally confirmed that MT-associated protein kinesin can alter the mechanical property of MTs, where increased interaction with kinesin was found to decrease the Young’s modulus (*E*) of MTs^37^. The inset in Fig. 5 shows the plot of 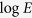 vs. 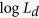 obtained from our previous study^37^ which reveals how interaction with kinesin, or in other words the spacing of kinesin affects the Young’s modulus of MT. To reproduce the dependence of Young’s modulus of MT on kinesin spacing, here we assume a fitting equation, equation (9), as shown below-


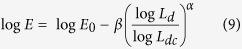


Here, 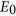
is the Young’s modulus of a bare MT, 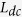 is the critical spacing between kinesins. The physical significance of 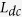 is that a MT is drastically softened if *L*_*d*_ is smaller than the 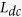. Using equation (9), we obtained the fitting curve with 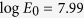, 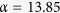, 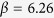, 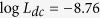 as shown in the inset of Fig. 5. Then in the light of equation (8) and equation (9), we brought a modification in the elastic foundation model by taking into consideration the role of kinesin in altering the mechanical property of MTs. As a result, modified equation for the elastic foundation model could be expressed by equation (10)-


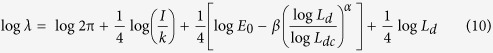


Figure 5 (main panel) shows the fitting curve according to equation (10), and as seen from this figure, at both low and high kinesin spacing regions the fitting curve according to equation (10) correlates well to our experimental results of MT buckling. From the fitting of our experimental results according to equation (10), we get equation (11) as-


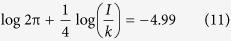


Using 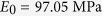 obtained from fitting of equation (9), and 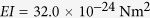 from literature^48^, we calculated the spring constant of kinesin, 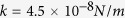 from equation (11). This value of spring constant for kinesin differs from that reported in literature^52^, reason of which is not clear at this moment and further investigation is required for confirmation. Moreover, using the critical wavelength of buckled MTs from our experiments we also calculated the critical buckling force for MTs at different kinesin spacing. At longer kinesin spacing, e.g. at 95 nm (for 10 nM kinesin concentration), the critical buckling force was ~1.6 pN, which increased to ~73.8 pN for the shorter kinesin spacing of 18 nm (for 200 nM kinesin concentration). This increasing trend of critical buckling force on decreasing the kinesin spacing and buckling wavelength is in a good agreement to that predicted by the elastic foundation model^32^, and recently reported computational predictions^53^. However, from the fitting curve the power dependence of critical buckling force, *F*_*c*_ on the kinesin spacing, *L*_*d*_ was found to be 4.13, instead of 0.5 predicted by the elastic foundation model which is shown in the Supplementary Information (Fig. S10). In spite of this difference, the values of critical buckling force for buckling of MTs experimentally determined in our work are comparable to those reported in different *in vitro* and *in vivo* studies^10^,^54^.

Finally we discuss the role of MT length in the deformation of MT under compression. As mentioned in the ‘Methods’ section, length of the MTs considered in our preceding discussions ranged from ~20 μm to ~50 μm and within this range no effect of MT length on buckling of MTs was noticed. As shown by the fluorescence microscopy images in the Supplementary Information (Fig. S11), buckling wavelength, and amplitude are found independent of the MT length. Now, Pampaloni et al., and Van den Heuvel et al., suggested that persistence length of a MT is dependent on its length^55^,^56^. Moreover, it is to consider that in cells MTs are 1–10 μm long, and in axons their length can be 50–100 μm^57^. Therefore, to investigate further, we focused on the buckling behavior of the MTs which are longer and shorter than those considered in our discussions so far. Here, for the cases of 50 and 200 nM kinesin concentrations we examined buckling of the MTs whose length ranged in ~12–75 μm, and ~5–72 μm respectively. As shown in the Supporting Information (Fig. S11), buckling wavelength, amplitude, and critical buckling force are still found independent of the MT length at both kinesin concentrations, even though we widened the range of length of MTs of our interest. Previously Jin et al., suggested that the critical buckling force and buckling wavelength are independent of MT length if the MT length exceeds a critical value^32^. Therefore, at this stage we focused on extremely short MTs; we furthered our examination of the buckling by taking into account the MTs which were shorter than ~10 μm and ~5 μm for the cases of 50 and 200 nM kinesin respectively. Our investigation revealed that critical strain for buckling of such short MTs differed from that of relatively longer MTs which is shown in the Supplementary Information (Fig. S12). For the case of 50 nM kinesin concentration, MTs with length ~7–10 μm underwent buckling at ~15% strain (number of MT considered, n = 22), whereas most of the MTs with length higher than ~12 μm were buckled at ~1.2% strain. Moreover, at this kinesin concentration, those MTs whose lengths were shorter than ~6–7 μm did not experience buckling even at 50% strain (n = 16). Similarly, for the 200 nM kinesin concentration, MTs with length ~4–5 μm underwent buckling at ~25% strain (n = 14), whereas most of relatively longer MTs buckled at ~2.0% strain. At this kinesin concentration, buckling was not observed for MTs whose lengths were shorter than ~3 μm (n = 21).

From this discussion it is now evident that the critical buckling force of short MTs is larger than that of relatively longer MTs, which coincides well with previous observations where longer MTs were found to bend or buckle easily compared to the shorter ones^20^,^58^,^59^. However, in order to understand the role of MT length in MT buckling, it appears important to investigate the buckling of extremely short MTs. Seemingly the buckling behavior of MTs becomes length independent when the MT length exceeds a certain value, i.e. the critical value. In this work, such critical length of MT appears to be ~10 μm and ~5 μm for the 50 and 200 nM kinesin respectively. Thus, the reason behind the observed length independence of MT buckling within the range of ~10–70 μm is now understandable. Moreover, the value of the critical length of MT is found to be dependent on the kinesin concentration, or in other word on the extent of interaction with kinesin. These observations are in a good agreement to the report by Jin et al. where critical buckling force of the MTs shorter than critical length were larger than that of relatively longer MTs^32^. Below the critical length of MT, an increase in critical buckling force on further decreasing the length of MT was also reported^32^. Moreover, decrease in critical length of MT on increasing the kinesin concentration as observed in our work could be supported by the previous report^32^. At present it is beyond the limit of this work to correlate the MT length, persistence length of MT, and buckling of MTs particularly in consideration of the role of kinesin in modulating the mechanical property of MTs^37^. A detailed and systematic investigation is required in future focusing on the buckling behavior of extremely short MTs (MTs with length shorter than the critical length), together considering the role of kinesin in modulating the mechanical property of MTs with different lengths.

Here, it is to emphasize that, the compression tests of MTs at different concentrations of kinesins were performed in the absence of ATP; thus the MT filaments were stationary and not under gliding motion during the compression tests. However, the buckled MTs started gliding on the kinesin coated PDMS substrate when ATP was applied to the system. Once the buckled MTs started gliding their buckling disappeared, which has already been discussed and shown in the Supplementary Information (Fig. S7). In this work, kinesins were attached to the PDMS substrate *via* interaction with anti-GFP antibody. Previously we determined the association constant between anti-GFP antibody and PDMS substrate, and compared the association constants of MT/kinesin, kinesin/anti-GFP antibody, and anti-GFP antibody/PDMS^37^. Although the interaction between anti-GFP antibody and PDMS substrate is the weakest one, we noticed no debonding or detachment of MTs from the PDMS substrate during the compression tests in this work.

In conclusion, we report the first ever compression stress induced buckling of MTs on a 2D elastic medium, that was demonstrated by using a newly developed ‘mechanical chamber system’. We investigated the effect of strain, strain rate, and most importantly the role of kinesin, a MT-associated protein, in the buckling of MTs. Applied strain was found to strongly affect the extent of buckling of MTs, but strain rate had no effect on the MT buckling. It is the density or spacing of the MT-associated protein that was found to determine the buckling mode of MTs. Comparison of our results with the predictions by the elastic foundation model suggests that any change in mechanical property of MTs by a MT-associated protein is required to consider for successfully predicting the buckling behavior of MTs. This work is expected to offer a new insight in understanding the role of surrounding medium or MT-associated proteins, strain etc. in the buckling of MTs, which consequently will play an indispensable role in obtaining a comprehensive understanding of the buckling of MTs in cell. The newly developed ‘mechanical chamber system’ is also expected to find potential applications in investigating the mechano-response of micro or nanoscale soft materials^24^,^60^ which would be significantly important from the viewpoint of biological or material science and nanotechnology.

## Additional Information

**How to cite this article**: Kabir, A. M. R. *et al.* Buckling of Microtubules on a 2D Elastic Medium. *Sci. Rep.* **5**, 17222; doi: 10.1038/srep17222 (2015).

## Supplementary Material

Supplementary Information
