# Supplementary material for: Buckling of Microtubules on a 2D Elastic Medium
Source: Sci Rep. 2015 Nov 24;5:17222. doi: 10.1038/srep17222 (PMC4657045; doi:10.1038/srep17222)
Supplement: Supplementary Information [file srep17222-s1.pdf]

Supplementary Information

Buckling of Microtubules on a 2D Elastic Medium

Arif Md. Rashedul Kabir[1], *Daisuke Inoue*[1], *Tanjina Afrin*,[2] *Hiroyuki Mayama*[3],

Kazuki Sada[1,2], *and Akira Kakugo*[1,2,*]

[1]Faculty of Science, Hokkaido University, Sapporo 060-0810, Japan

[2]Graduate School of Chemical Sciences and Engineering, Hokkaido University, Sapporo 060-0810, Japan

[3]Department of Chemistry, Asahikawa Medical University, Asahikawa 078-8510, Japan

*To whom correspondence should be addressed

E-mail: kakugo@sci.hokudai.ac.jp

Telephone/fax: +81-11-706-3474

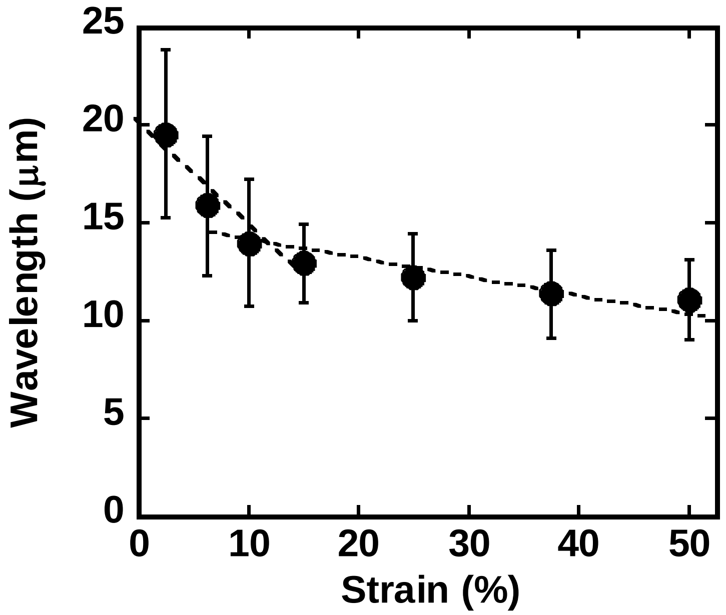

Figure S1: Representative graph showing the adopted principle for selecting wavelength for verification of the elastic foundation model. This result is obtained from experiments using 10 nM kinesin. As shown in this figure, change of wavelength of buckled MTs on compression depends on the strain applied. The change of wavelength at low strain region is much bigger (steeper line) than that at relatively high strain region. Thus, wavelength corresponding to the intersection of the two straight lines as drawn here was selected for verifying the elastic foundation model. Error bar: standard deviation.

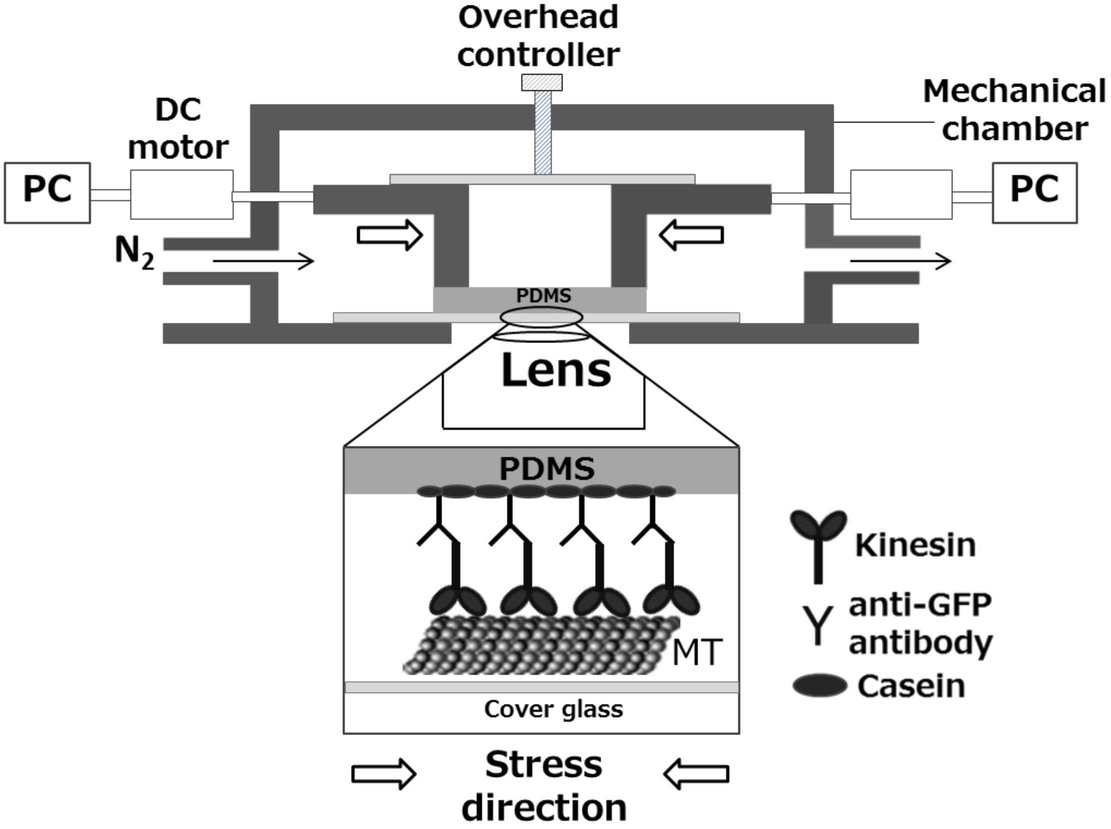

Figure S2: Schematic diagram showing the design of the 'mechanical chamber system' that was used to demonstrate the compression stress induced buckling of MTs on a 2D elastic medium.

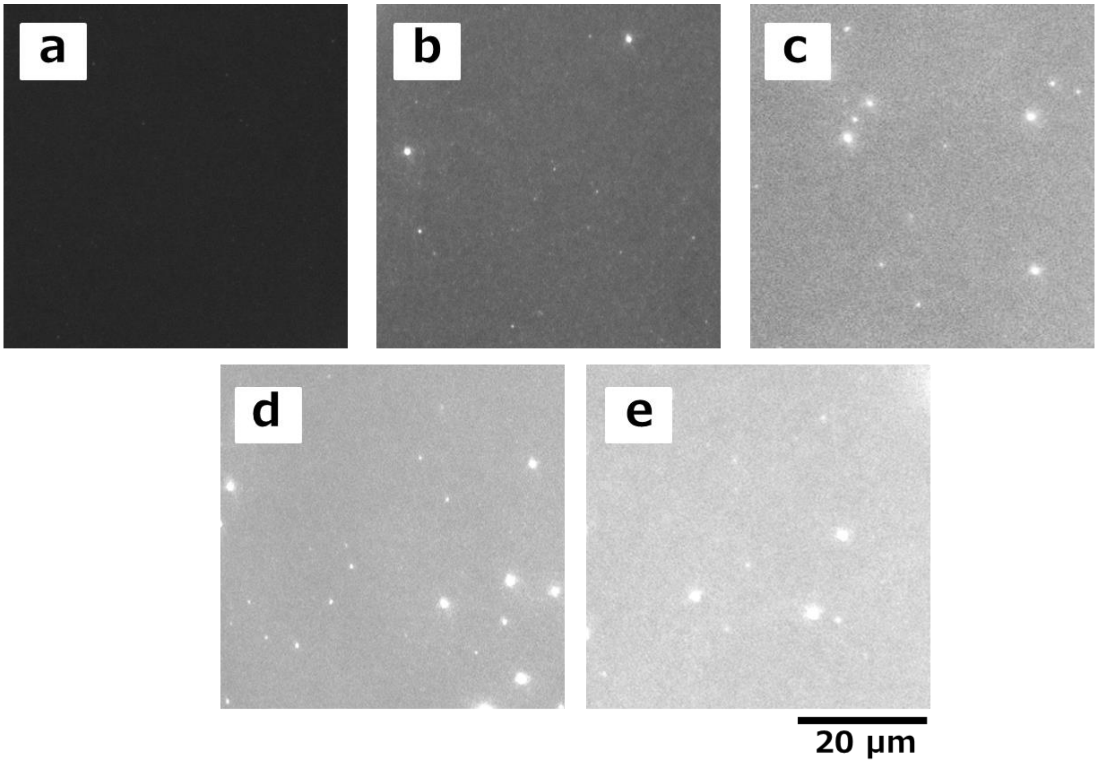

Figure S3: Change in fluorescence intensity on changing the kinesin concentration in the compression tests of MTs. Concentration of kinesin used were: (a) 10, (b) 30, (c) 50, (d) 100 and (e) 200 nM. Increased kinesin concentration resulted in higher density of the kinesins on PDMS substrate that was confirmed by the measurements using QCM (see the ‘Methods’), and also evident from the increased fluorescence intensity as shown here by the fluorescence microscopy images.

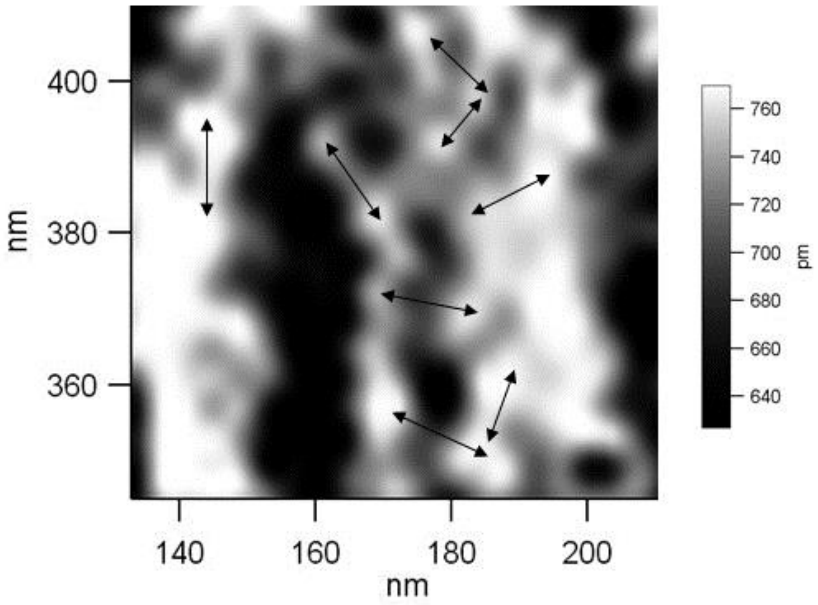

Figure S4: Atomic force microscopy (AFM) image of kinesins attached to the PDMS substrate. This image was captured for the kinesin concentration of 200 nM. From this AFM image, the inter-kinesin spacing (shown by the black arrows) was measured 12.77±1.0 nm, which is well comparable to that estimated by QCM (~18 nm).

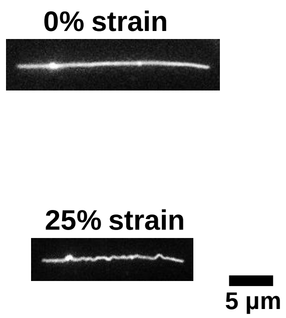

Figure S5: Fluorescence microscopy images showing compression stress induced buckling of a MT, where the compression test was performed using 300 nM kinesin. At such a high kinesin concentration, buckling crests of the deformed MTs were difficult to identify and separate from each other which rendered the MTs unsuitable for any quantitative characterization. For this reason, results of MT compression tests performed using 300 nM kinesin could not be considered.

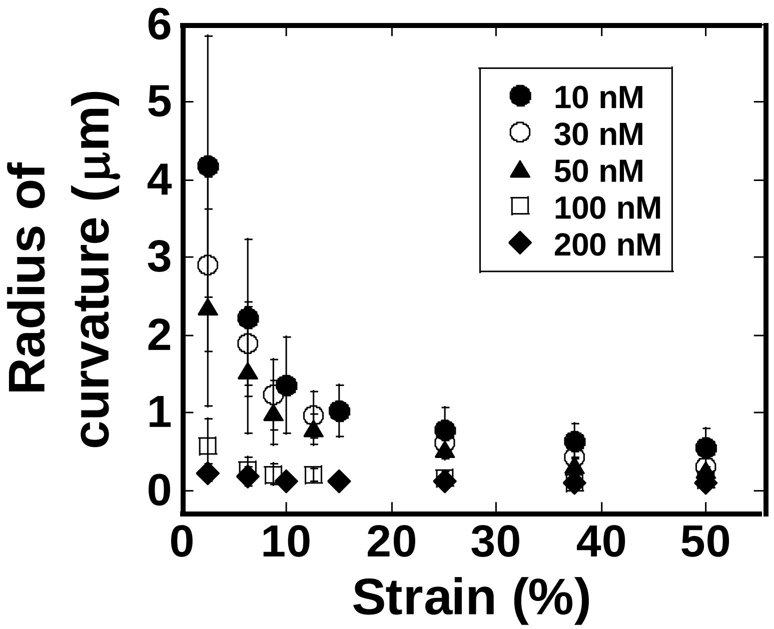

Figure S6: Change in radius of curvature of buckled MTs on changing the compression strain at different kinesin concentrations as shown in the inset.

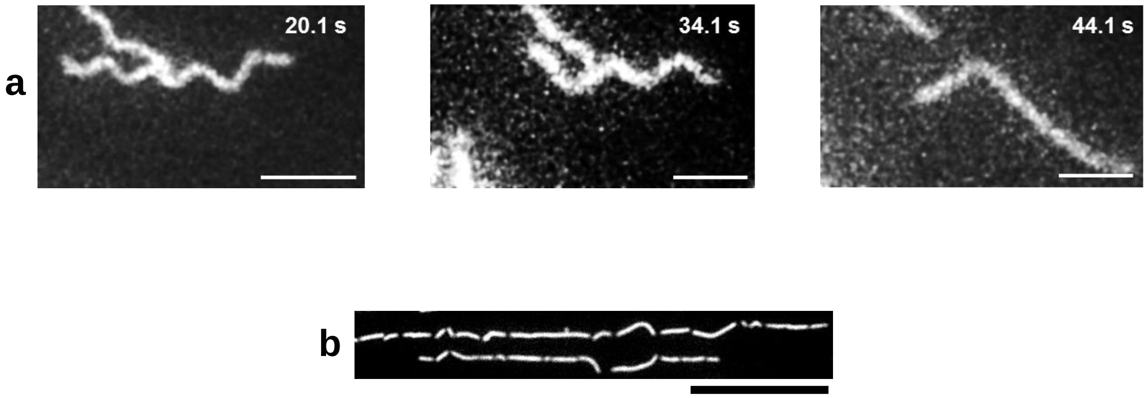

Figure S7: (a) Time lapse images showing what happened when a buckled MT was exposed to adenosine triphosphate (ATP). As soon as the ATP buffer was added to the system (left image), the buckled MT started gliding on the kinesin coated PDMS substrate, adopted a straight morphology and soon the buckling disappeared (right image). The MT was buckled under application of 50% compression strain at a rate of 0.42% s^{-1}, and the experiment was performed using 50 nM kinesin. (b) Breakage of MTs which were buckled under 50% compression strain at 200 nM kinesin. The fracturing of MTs was clearly visible on removal of the applied compression strain. Scale bar: 5 µm.

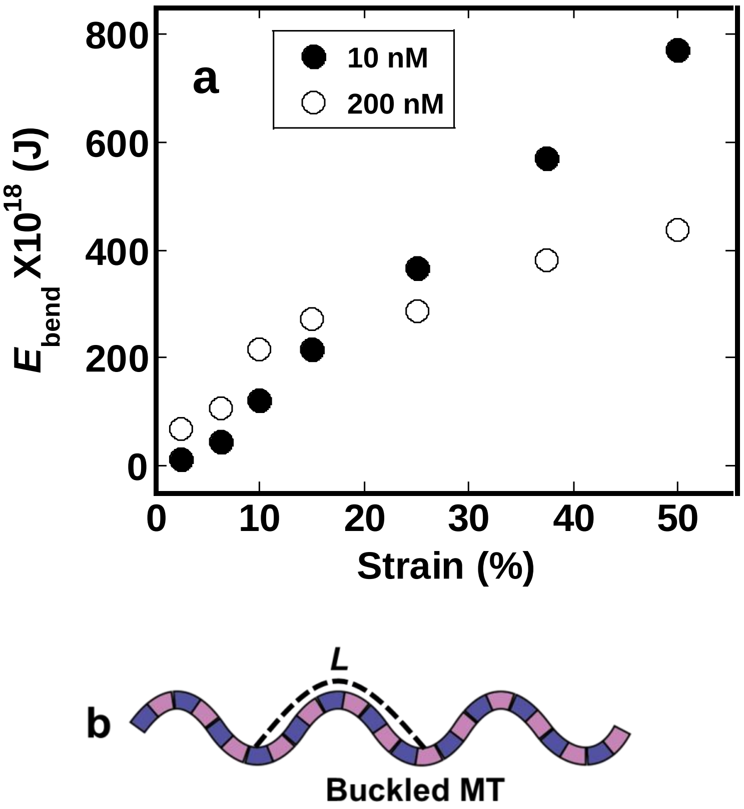

Figure S8: (a) Change in bending energy E_{bend} stored in the buckled MTs on changing compression strain at the lowest (10 nM), and highest (200 nM) kinesin concentration. The bending energy was calculated using the equation $E_{\text{bend}} = \frac{EIL}{2R^2}$. Here, E is the Young's modulus, and I is the second moment of inertia of MT, L is the contour length of MT along a single buckling crest and R is radius of curvature of MT. To calculate the E_{bend} we considered I=32.82×10^{-32} m^4; and E=49.75 and 7.15 MPa for 10 and 200 nM kinesin respectively. The definition of L is shown schematically in (b).

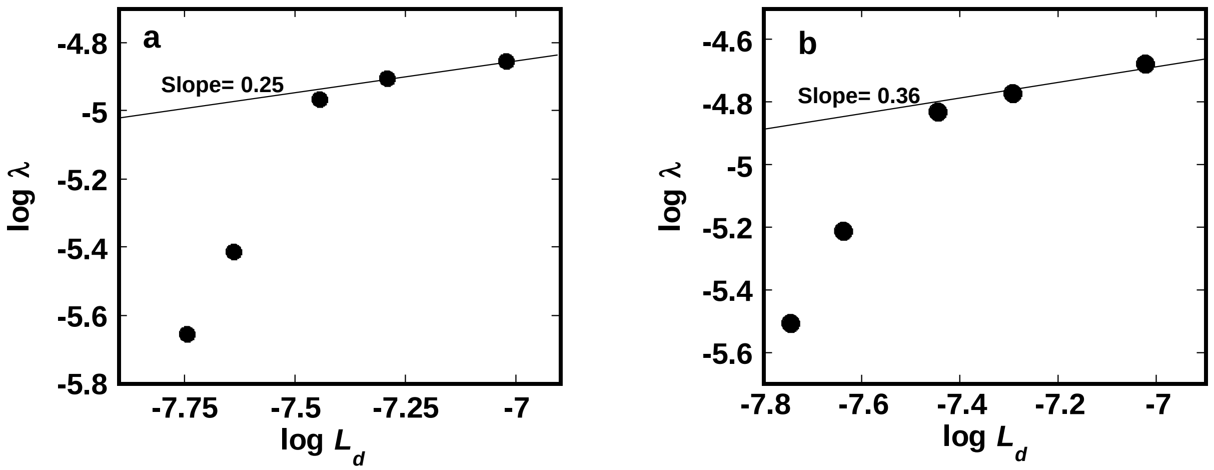

Figure S9: Verification of the elastic foundation model where the buckling wavelengths (λ) were selected by the intersection method (a), as shown in Figure S1 and discussed in 'Methods', and from the critical buckling strain (b). In both cases, experimental results were in good agreement at long kinesin spacing regions (low kinesin density regions), but significantly deviated at short kinesin spacing regions (high kinesin density regions). Here, L_d stands for the kinesin spacing on the PDMS substrate.

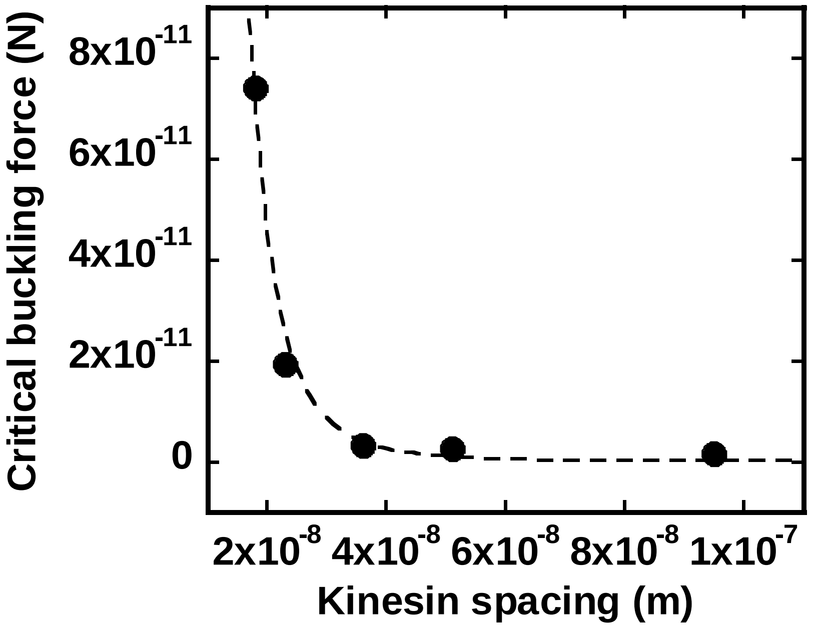

Figure S10: Change of critical buckling force with the change of kinesin spacing. As the kinesin spacing increased, the critical buckling force was found to decrease which is in agreement to the elastic foundation model. The curve fitting was performed according to the equation $F_{cr} = 2\sqrt{k.EI}.\frac{1}{\sqrt{L_d}}$ where, F_{cr} is the critical buckling force and L_d is the kinesin spacing, k is the spring constant of kinesin and EI is the bending rigidity of MTs. The values of EI= 9.03×10^{-24} Nm^2 and k= 0.65×10^{-3} Nm^{-1} were considered for curve fitting.

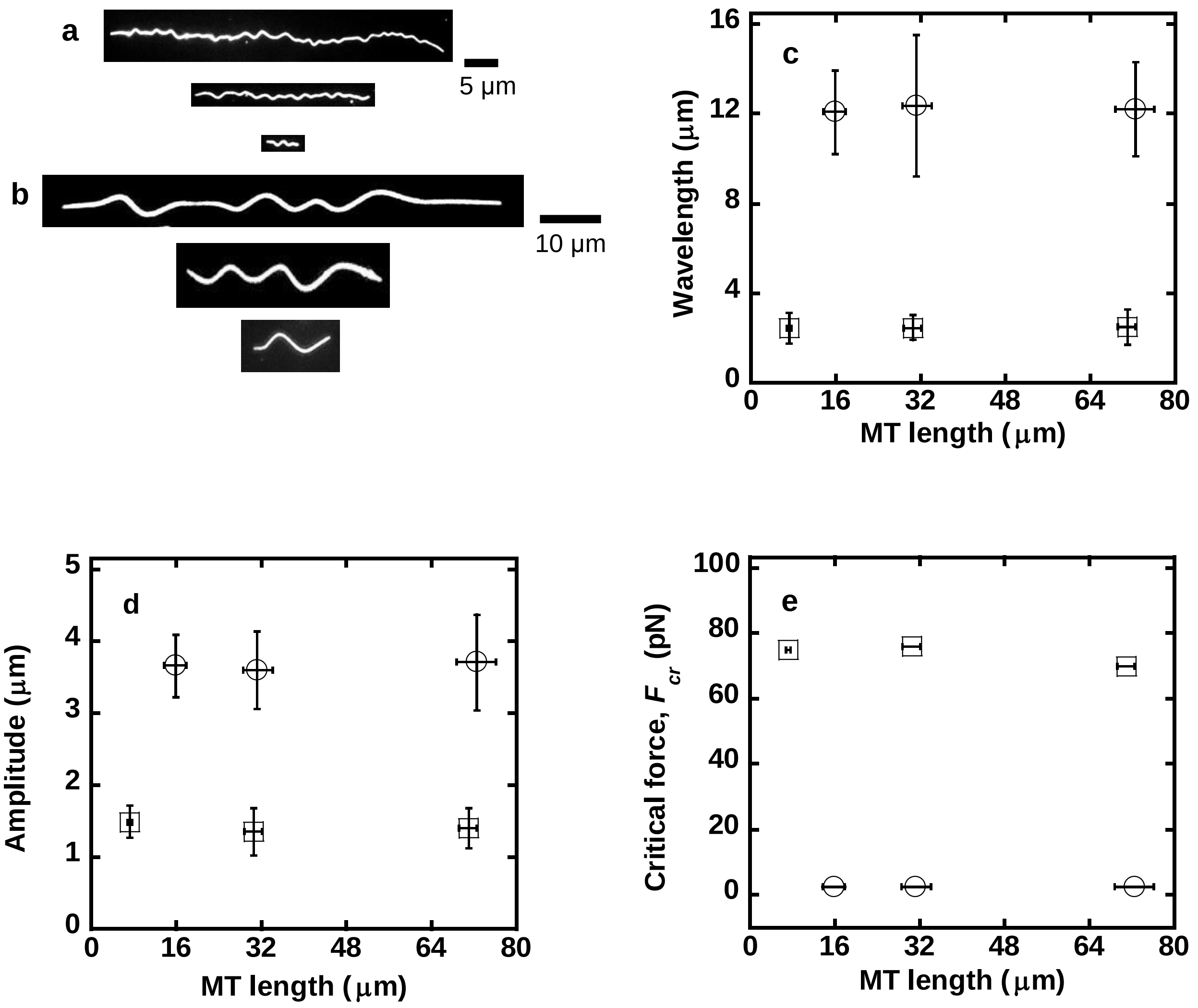

Figure S11: Fluorescence microscopy images (captured at 12.5% strain) showing effect of MT length on buckling at two different kinesin concentrations: (a) 200 nM, and (b) 50 nM. MT buckling was found independent of the MT length at both kinesin concentrations which is in agreement to the elastic foundation model. Buckling wavelength (c), amplitude (d) and critical force for buckling (e) were also found independent of the MT length over a wide range. In (c), (d) and (e) the symbols 'circle' and 'square' represent 50 and 200 nM kinesin concentration respectively. Error bar: standard deviation.

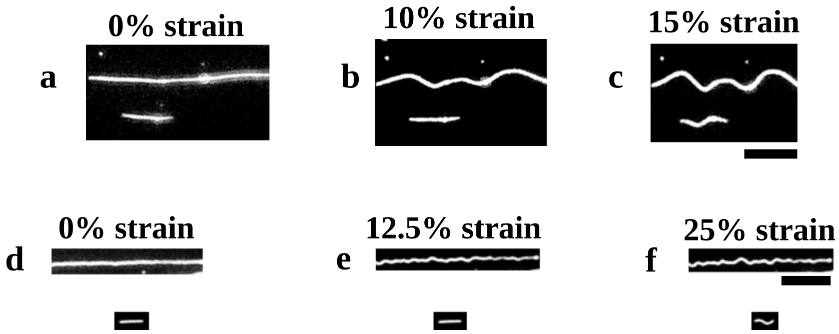

Figure S12: Effect of MT length on the critical buckling strain. The images were captured for two different kinesin concentrations of 50 nM (a-c), and 200 nM (d-f). At 50 nM kinesin concentration, a MT with length less than 10 µm buckled at 15% strain, although a relatively longer MT with length ~30 µm and most of the MTs longer than ~12 µm buckled at 1.2% strain. Similarly, At 200 nM kinesin concentration, a MT with length close to ~5 µm buckled at 25% strain, although a relatively longer MT with length ~20 µm and most of the longer MTs buckled at 2.0% strain. Scale bars: 10 µm (a-c) and 5 µm (d-f).
